# Male Sex is Associated with a Reduced Alveolar Epithelial Sodium Transport

**DOI:** 10.1371/journal.pone.0136178

**Published:** 2015-08-20

**Authors:** Till Kaltofen, Melanie Haase, Ulrich H. Thome, Mandy Laube

**Affiliations:** Center for Pediatric Research Leipzig, Division of Neonatology, Hospital for Children & Adolescents, University of Leipzig, Leipzig, Germany; University of Geneva, SWITZERLAND

## Abstract

Respiratory distress syndrome (RDS) is the most frequent pulmonary complication in preterm infants. RDS incidence differs between genders, which has been called the male disadvantage. Besides maturation of the surfactant system, Na^+^ transport driven alveolar fluid clearance is crucial for the prevention of RDS. Na^+^ transport is mediated by the epithelial Na^+^ channel (ENaC) and the Na,K-ATPase, therefore potential differences in their expression or activity possibly contribute to the gender imbalance observed in RDS. Fetal distal lung epithelial (FDLE) cells of rat fetuses were separated by sex and analyzed regarding expression and activity of the Na^+^ transporters. Ussing chamber experiments showed a higher baseline short-circuit current (I_SC_) and amiloride-sensitive ΔI_SC_ in FDLE cells of female origin. In addition, maximal amiloride-sensitive ΔI_SC_ and maximal ouabain-sensitive ΔI_SC_ of female cells were higher when measured in the presence of a permeabilized basolateral or apical membrane, respectively. The number of FDLE cells per fetus recoverable during cell isolation was also significantly higher in females. In addition, lung wet-to-dry weight ratio was lower in fetal and newborn female pups. Female derived FDLE cells had higher mRNA levels of the ENaC- and Na,K-ATPase subunits. Furthermore, estrogen (ER) and progesterone receptor (PR) mRNA levels were higher in female cells, which might render female cells more responsive, while concentrations of placenta-derived sex steroids do not differ between both genders during fetal life. Inhibition of ER-β abolished the sex differences in Na^+^ transport and female cells were more responsive to estradiol stimulation. In conclusion, a higher alveolar Na^+^ transport, possibly attributable to a higher expression of hormone receptors in female FDLE cells, provides an explanation for the well known sex-related difference in RDS occurrence and outcome.

## Introduction

**Fig 1:**
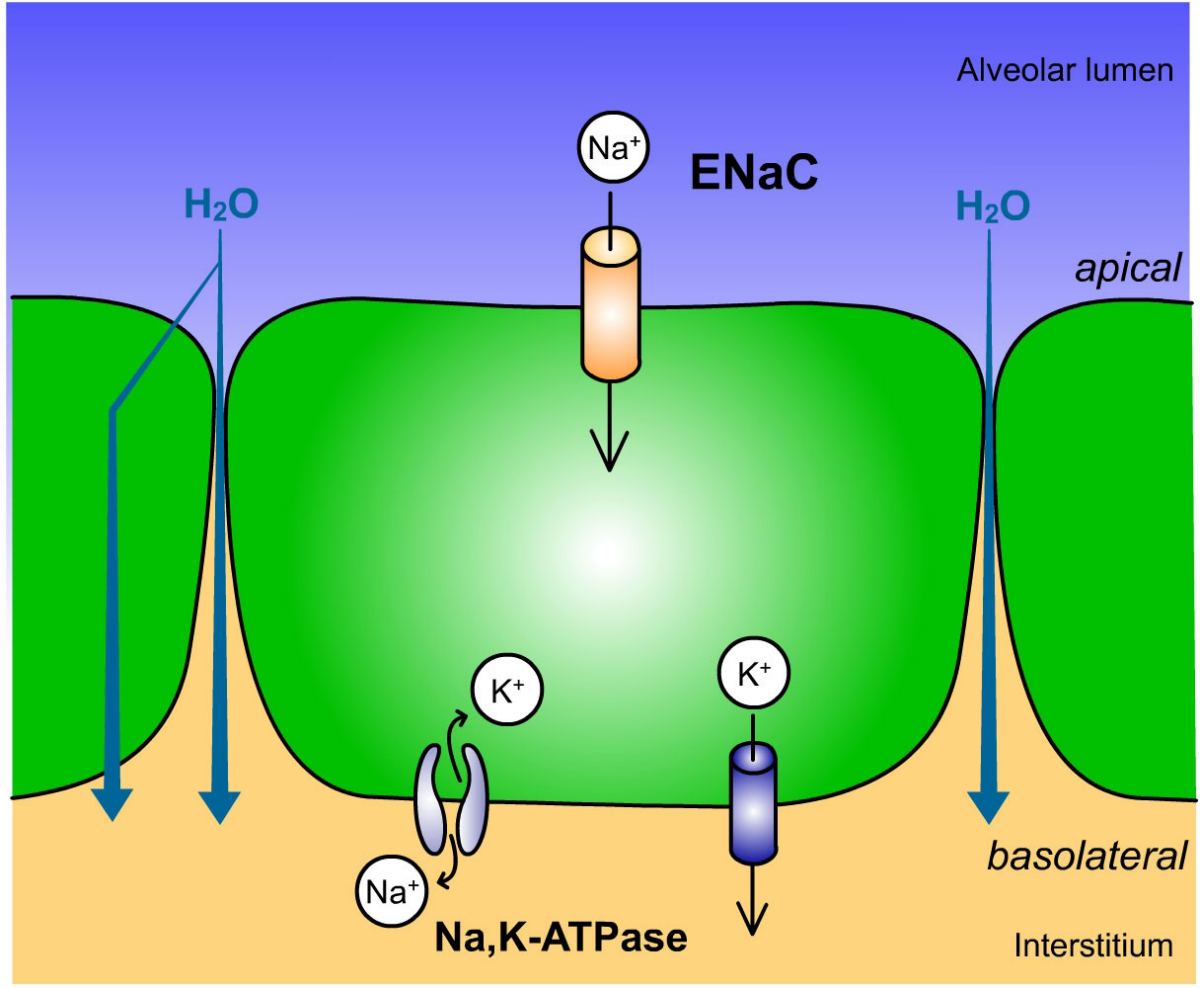
Alveolar epithelial Na^+^ transport. Na^+^ enters the apical membrane of alveolar epithelial cells through ENaC and is actively extruded by the Na,K-ATPase in the basolateral membrane compartment, whereas K^+^ ions are recycled by K^+^ channels. Thereby pulmonary fluid is absorbed as it passively follows the osmotic gradient generated by vectorial Na^+^ transport.

Respiratory distress syndrome (RDS), also known as hyaline membrane disease, is the leading cause of morbidity and mortality in preterm infants [1]. Surfactant deficiency is known as a causative factor for RDS development [2,3]. However, with increasing gestational age, relevance of surfactant deficiency diminishes while impairment of the lung to absorb alveolar fluid becomes more important [4]. Alveolar fluid clearance (AFC) is driven by unidirectional Na^+^ transport across the alveolar epithelia [5]. In this process Na^+^ enters the apical membrane of alveolar type II (ATII) cells through epithelial Na^+^ channels (ENaC) and is actively extruded basolaterally by the Na,K-ATPase [6] (Fig 1). ENaC consists of three homologous subunits α-, β- and γ-ENaC [7], while the Na,K-ATPase in ATII cells is composed of α_1_- and β_1_-subunits [8]. Water follows the resulting osmotic gradient passively from the airspaces to the pulmonary interstitium and is thereby absorbed into the systemic circulation. Disturbances of alveolar epithelial Na^+^ transport result in pulmonary fluid accumulation, which compromises gas exchange and represents another causative factor contributing to RDS in preterm infants [9,10]. An overall reduction in epithelial Na^+^ transport was demonstrated in preterm infants, that was associated with RDS development [11], and survival of adult patients with acute RDS was related to AFC efficiency [12]. A general delay in lung maturation has been described in male fetuses [13] and male sex represents a major risk factor for RDS occurrence with a sex ratio of 1:1.7 [2,14,15]. Antenatal glucocorticoid treatment improves postnatal lung function by several effects, including increased surfactant production and increased AFC, but was not able to abolish the gender imbalance [16]. Sex differences in alveolar Na^+^ transport have not been addressed in preterm lungs. Female sex steroids have been shown to stimulate alveolar Na^+^ transport *in vitro* [17–20] and prenatal estrogen and progesterone deprivation reduced amiloride-sensitive AFC *in vivo* [21]. In addition, newborn infants suffering from RDS showed diminished estrogen [22] and progesterone plasma concentrations [23] in comparison to infants of the same gestational age without RDS. Estrogen substitution in a randomized trial resulted in significantly less respiratory distress and higher survival in preterm infants [24]. Moreover, a sexual dimorphism in ENaC expression has been described in adult rats and ENaC mRNA expression was further increased by estradiol (E2) and progesterone *in vivo* [25]. However, since estrogen and progesterone plasma concentrations are similar in male and female fetuses [26,27], female sex steroid levels *per se* are not likely the cause for sex differences in fetal lung development. Therefore, the aim of this study was to analyze putative sex-related differences in Na^+^ transport across fetal alveolar epithelia and elucidate mechanisms regulating potential sex differences in alveolar Na^+^ transport.

## Materials and Methods

### Cell isolation and culture

Animal care and experimental procedures were approved by the institutional review board (IRB: Landesdirektion Leipzig, permit number: T36/13). Sprague-Dawley rats were housed at the Medical Experimental Center (MEZ) of Leipzig University. Rodents were maintained in rooms with a 12 h light-dark cycle, constant temperature (22°C) and humidity (55%). Food and water were available *ad libitum*. Pregnant rats were euthanized by carbon dioxide inhalation and E20-21 gestational day fetal lungs (term = E22) were used for cell isolation. Fetal rats were separated by sex through visual determination of the anogenital distance [28,29]. Validation of visual sex determination was done by PCR-amplification of the SRY gene.

Isolation of fetal distal lung epithelial (FDLE) cells, a model of preterm ATII cells, was performed as described previously [20,30,31]. For each gender a separate cell isolation procedure was done in parallel, and equal numbers of male and female fetuses were used from each litter. Briefly, lungs were mechanically dissociated and digested in a solution containing 0.125% trypsin (Life Technologies, Darmstadt, Germany) and 0.4 mg/ml DNAse (CellSystems, Troisdorf, Germany) in Hanks’ Balanced Salt Solution (HBSS) (Life Technologies) for 10 min at 37°C. Digestion was stopped by addition of Minimum Essential Eagle's Medium (MEM) with 10% fetal bovine serum (FBS) (Biochrom, Berlin, Germany). Then cells were centrifuged (440 x g) and resuspended in 15 ml MEM containing 0.1% collagenase (CellSystems) and DNAse. After an incubation period of 15 min at 37°C, digestion was stopped by adding 15 ml MEM containing 10% FBS. To remove contaminating fibroblasts, cells were plated twice for one hour at 37°C in cell culture flasks. The cell culture supernatant contained epithelial cells with > 95% purity [30]. Before seeding, cell numbers were determined microscopically using a Neubauer chamber. Thereby, FDLE cell number yields per fetus were calculated for each sex. For Ussing chamber experiments, cells were seeded on permeable Snapwell inserts with a surface area of 1.1 cm^2^ (Costar # 3407, Corning Inc., NY) at a density of 10^6^ cells per insert. For mRNA expression analysis, cells were seeded on permeable Transwell inserts with a surface area of 4.6 cm^2^ (Costar # 3412) at a density of 2 x 10^6^ cells per insert. In addition, direct lysis of 2 x 10^6^ freshly isolated cells was done to determine how cell culture influences mRNA expression. Cells were cultured under submerged conditions with culture medium containing MEM with 10% FBS, glutamine (2 mM, Life Technologies), penicillin (100 U/ml, Life Technologies), streptomycin (100 μg/ml, Life Technologies) and amphotericin B (0.25 μg/ml, Life Technologies). Cell culture media was changed daily. To analyze responsiveness to E2 (Sigma-Aldrich) FDLE cells were cultured in serum-free complete medium (Cellgro, Mediatech, Herndon, VA) supplemented with different E2 concentrations for 24 h. Cells of female and male origin were always derived from the same litter, treated equally and recorded simultaneously.

### Ussing chamber measurements

Experiments were performed four days after seeding of the cells. Only measurements with a transepithelial resistance (R_te_) exceeding 300 Ω cm^2^ were included in the data analyses. Ussing chambers were filled with a solution containing: 145 mM Na^+^, 5 mM K^+^, 1.2 mM Ca^2+^, 1.2 mM Mg^2+^, 125 mM Cl^-^, 25 mM HCO_3_^-^, 3.3 mM H_2_PO_4_^-^ and 0.8 mM HPO_4_^2-^ (pH 7.4). The basolateral compartment contained 10 mM glucose whereas 10 mM mannitol was used in the apical compartment to avoid a possible contribution of a putative apical Na^+^-glucose co-transporter [32]. Both chambers were continuously bubbled with a mixture of 5% CO_2_ and 95% O_2_. Equivalent short-circuit currents (I_SC_) were assessed every 20 s by measuring transepithelial potential difference (V_te_) and R_te_ with a transepithelial current clamp (Physiologic instruments, San Diego, CA) and calculating the quotient I_SC_ = V_te_/R_te_. After the baseline I_SC_ reached a stable plateau (I_base_), amiloride (10 μM, # A7410, Sigma-Aldrich, Taufkirchen, Germany) was added to the apical compartment to determine the amiloride-sensitive ΔI_SC_ (I_amil_) as a measure of ENaC activity.

To determine the maximal Na,K-ATPase activity, the apical membrane was permeabilized with amphotericin B (a pore forming antibiotic, 10 μM, # A-4888, Sigma-Aldrich), loading the cytosol with Na^+^ and thereby enabling the Na,K-ATPase to exhibit its maximum Na^+^ transport capacity [20,31,33]. I_SC_ was measured every 5 s with a transepithelial voltage clamp. When I_SC_ had risen to its maximum value, 1 mM ouabain (# O3125, Sigma-Aldrich) was added to the basolateral compartment and the ouabain-sensitive component of the amphotericin-induced maximal I_SC_ (ouab_max_) was calculated. Furthermore, apical Na^+^ permeability undisturbed of the Na,K-ATPase was determined by adding amphotericin B (100 μM) to the basolateral compartment. For this experimental set-up 140 mM of basolateral Na^+^ was replaced by 116 mM *N*-methyl-D-glucamine (NMDG^+^, # M-2004, Sigma-Aldrich) and 24 mM choline, generating a 145:5 apical-to-basolateral Na^+^ gradient. Following amphotericin B addition, all I_SC_ is due to passive Na^+^ flux through apical Na^+^ conductive pathways down the Na^+^ gradient from the apical to the basolateral side [34]. When I_SC_ had reached its maximum value, the amiloride-sensitive component (amil_max_) was determined by adding 10 μM amiloride to the apical compartment. Amiloride and ouabain were prepared in water. The ER-β inhibitor 4-[2-Phenyl-5,7-*bis*(trifluoromethyl)pyrazolo[1,5-*a*]pyrimidin-3-yl]phenol (PHTPP, # Cay16025-5, Biomol, Hamburg, Germany) was diluted in dimethyl sulfoxide (DMSO, # 472301, Sigma-Aldrich) and FDLE cells treated with 20 μM PHTPP [35,36] for 24 h prior to Ussing chamber analyses. Control cells were treated with the respective solvent to exclude an influence on the evoked responses.

### mRNA expression analysis

Sampling of mRNA specimens was done at two different time points, directly after cell isolation and after four days in culture to match the time of the Ussing chamber measurements. RNA was isolated with the Purelink RNA Mini Kit (Life Technologies) and treated with DNAse I (Life Technologies) according to the manufacturer’s instructions. Then reverse transcription was performed with pre-annealing of 1 μg RNA with Oligo(dT)_18_ primers (ThermoFisher Scientific, St. Leon-Rot, Germany), followed by the addition of Superscript III (Life Technologies) and incubation at 55°C for 1 h and 75°C for 15 min. The cDNA was diluted 1:10 in Tris-EDTA-buffer (AppliChem, Darmstadt, Germany). Real-time quantitative PCR (RT-qPCR) was conducted in the CFX 96 Real-Time system (Bio-Rad, Munich, Germany) using SYBR-Green (Molecular Probes, Eugene, OR) with the Platinum *Taq* polymerase (Life Technologies) and gene-specific primers (Table 1). RT-qPCR runs were initiated with 95°C for 3 min, followed by 40 cycles at 95°C for 30 s, primer annealing at 60°C for 30 s and primer elongation at 72°C for 30 s. Within each RT-qPCR run 3–4 biological replicates and 3 technical replicates were used and results confirmed with two additional FDLE cell preparations. Absolute quantification was performed using a serial dilution of target-specific plasmid DNA as internal standard curve. PCR efficiencies were calculated by standard curve analysis and standard curve plots showed a high coefficient of determination (*R* ^2^>0.99). The resulting molecule concentrations were normalized to a reference gene encoding for the 60S ribosomal protein L13a (Rpl13a) [37]. Constant expression of Rpl13a was confirmed against other common reference genes. Fold change of mRNA levels was calculated with the relative standard curve method and expressed in fold change of control (male). To determine the specificity of the PCR reaction melting curves and gel electrophoresis of PCR products were routinely performed.

**Table 1:** Primer sequences.

Gene	Primer (forward, 5'- 3')	Primer (reverse, 3'- 5')
**α-*ENaC***NM_031548.2	TTCTGGGCGGTGCTGTGGCT	GCGTCTGCTCCGTGATGCGG
**β-*ENaC***NM_012648.1	TGCAGGCCCAATGCCGAGGT	GGGCTCTGTGCCCTGGCTCT
**γ-*ENaC***NM_017046.1	CACGCCAGCCGTGACCCTTC	CTCGGGACACCACGATGCGG
***Na*,*K-ATPase*-α**_**1**_NM_012504.1	GGACGAGACAAGTATGAGCCCGC	CATGGAGAAGCCACCGAACAGC
***Na*,*K-ATPase*-α**_**2**_ [38]NM_012505.2	GCTCTGGGCGGCTTCTTCACC	GATCTTGTTCTTCATGCCCTGCTGG
***Na*,*K-ATPase*-β**_**1**_NM_013113.2	GCGCAGCACTCGCTTTCCCT	GGGCCACACGGTCCTGGTACG
***ER-*β**NM_012754.1	CTGGTGAGCCGTCCCAGCATG	GGTGGTCGATGGAGCGCCAC
***PR-A/B*** [39]NM_022847.1	GGCAAATCCCACAGGAGTTTGTC	CAGACATCATTTCCGGAAATTCC
***PDGF-A*** [40]NM_012801	AAGCATGTGCCGGAGAAGCG	GCTCCTCTAACCTCACCTGGACC
***Rpl13a***NM_173340.2	GGGCCATCTTCTGGGCCGC	CATGCCTCGCACAGTGCGCC

### Measurement of lung wet-to-dry weight ratio

Lung wet-to-dry (W/D) weight ratio was used as an index of lung water content and determined as described previously [9,41]. In brief, lungs from male and female fetuses (E21) and newborn pups (P0) were excised, placed in HBSS and left lung lobes were dissected free. Lung lobes were blotted on filter paper to remove excess liquid, weighed wet, dried overnight at 80°C and reweighed. Lung W/D weight ratio was calculated by dividing the wet by the dry weight.

### Statistical analysis

Statistical analysis was performed with GraphPad Prism software (GraphPad Software, La Jolla, CA) and significant differences between female and male study groups were determined by unpaired T-test or analysis of variance (ANOVA) followed by Dunnett’s *post hoc* test, as appropriate. A probability of p<0.05 was considered significant for all statistical analyses.

## Results

### Ussing chamber measurements

For electrophysiological measurements, 385 FDLE cell monolayers were obtained from 13 different cell isolations. 372 monolayers had a R_te_ > 300 Ω cm^2^ and were included in the analysis. Their mean R_te_ was 998.7 ± 472.4 Ω cm^2^ (mean ± SD).

**Fig 2:**
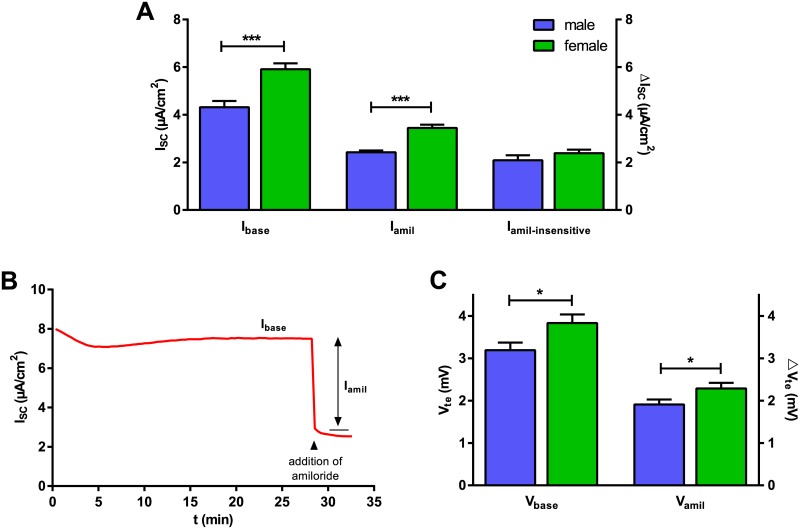
Female FDLE cells display an increased Na^+^ transport. (A) I_base_ was the I_SC_ after mounting the monolayers in the Ussing chambers, the amiloride-sensitive (I_amil_) current represents the current reduction induced by amiloride (10 μM). I_base_ and I_amil_, but not the amiloride-insensitive I_SC_, were significantly higher in FDLE cells of female origin (n = 45/56; mean + SEM; ***p<0.001 by T-test). (B) Representative current tracing of FDLE monolayers. (C) V_base_ and V_amil_ were significantly higher in FDLE cells of female origin (n = 45/56; mean + SEM; *p<0.05 by T-test).

Sex-related differences in I_base_ and I_amil_ were assessed on intact monolayers of male and female FDLE cells. I_base_ showed significantly higher values in FDLE cells of female origin compared with male (mean ± SEM ♂: 4.3 ± 0.3 to ♀: 5.9 ± 0.2 μA/cm^2^; p<0.001) (Fig 2A). Likewise, I_amil_ of female monolayers was significantly higher in comparison with monolayers of male origin (♂: 2.4 ± 0.1 to ♀: 3.4 ± 0.1 μA/cm^2^; p<0.001) (Fig 2A). In contrast, the amiloride-insensitive I_SC_, likely representing Cl^-^ transport, was not significantly different between male and female derived monolayers (♂: 2.1 ± 0.2 and ♀: 2.4 ± 0.1 μA/cm^2^; p>0.05) (Fig 2A). A current tracing is shown in Fig 2B. The observed currents were approximately 40% higher in female cells compared with male. Higher I_base_ and I_amil_ in females were reflected by a higher V_te_ in monolayers of female origin. Baseline V_te_ (V_base_) was significantly higher in FDLE cells of female origin compared with male (mean ± SEM ♂: 3.2 ± 0.2 to ♀: 3.8 ± 0.2 mV; p<0.05) as was the amiloride-sensitive V_te_ (V_amil_) (mean ± SEM ♂: 1.9 ± 0.1 to ♀: 2.3 ± 0.1 mV; p<0.05) (Fig 2C).

**Fig 3:**
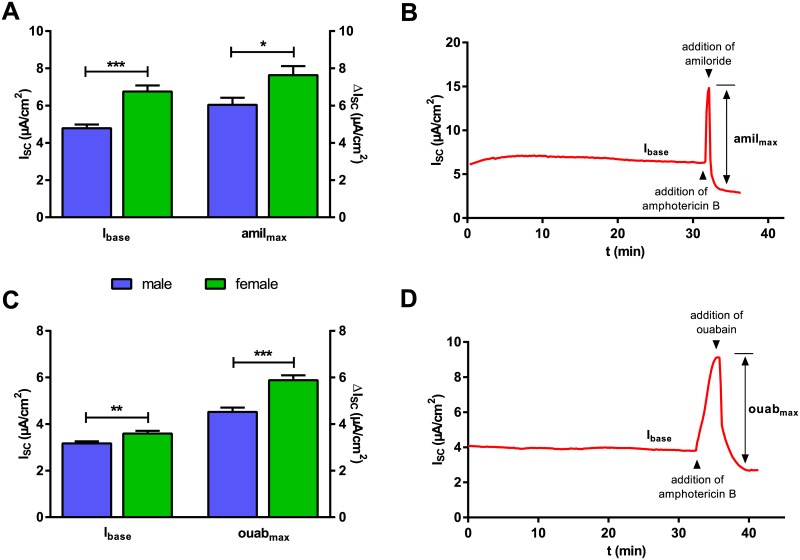
Female FDLE monolayers show a higher ENaC and Na,K-ATPase activity in permeabilized membrane measurements. After the I_SC_ reached a plateau (I_base_), amphotericin B was used to permeabilize either the basolateral (100 μM) or the apical (10 μM) membrane. At the maximal current increase either amiloride (10 μM) or ouabain (1 mM) were applied and amil_max_ (A) or ouab_max_ (C) determined. I_base_, amil_max_ and ouab_max_ were significantly higher in FDLE cells of female origin (A: n = 35/32; C: n = 53/51; mean + SEM, *p<0.05, **p<0.01 and ***p<0.001 by T-test). (B/D) Representative current tracings of permeabilized membrane measurements.

In a second set of electrophysiological measurements, apical Na^+^ conductance and basolateral active transport capacity were measured separately. As in the previous experiment, marked sex-related differences in I_base_ were also observed. Amil_max_ measured in the presence of a 145:5 apical-to-basolateral Na^+^ gradient and permeabilized basolateral membrane was significantly higher in FDLE cells of female origin compared with male (♂:6.0 ± 0.4 to ♀: 7.6 ± 0.5 μA/cm^2^; p< 0.05) (Fig 3A). A typical I_SC_ tracing for this experimental set up is shown in Fig 3B. Ouab_max_ also showed a significantly higher current in FDLE cells of female origin compared with male (♂: 4.5 ± 0.2 to ♀: 5.9 ± 0.2 μA/cm^2^; p<0.001) (Fig 3C). A typical I_SC_ tracing for this experimental set up is shown in Fig 3D.

### mRNA expression analysis

**Fig 4:**
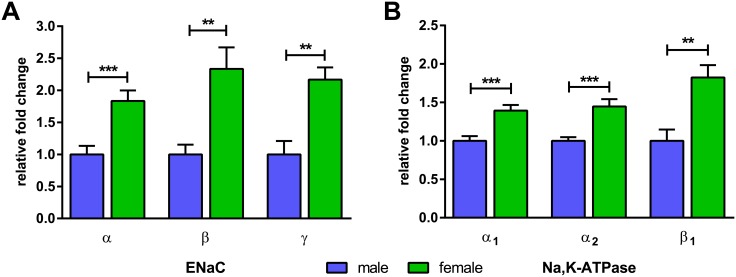
mRNA expression of ENaC and Na,K-ATPase subunits is higher in female FDLE cells. mRNA expression of (A) α-ENaC, β-ENaC and γ-ENaC and (B) Na,K-ATPase-α_1_, Na,K-ATPase-α_2_ and Na,K-ATPase-β_1_ was significantly higher in female compared with male FDLE cells after 4 days in culture (n = 7–12; mean + SEM; **p<0.01 and ***p<0.001 by T-test).

Analysis of mRNA expression revealed different expression patterns for the Na^+^ transporters and hormone receptors in FDLE cells of female and male origin. Measurements after 4 days in culture matched the Ussing chamber experiments. The ENaC subunits α, β and γ all showed a significantly higher mRNA level in female FDLE cells. More precisely, the α-ENaC subunit mRNA level in female cells was 1.8-fold higher than in male cells (p<0.001) (Fig 4A). Furthermore, mRNA levels of β- and γ-ENaC were up to 2.3- and 2.2-fold higher, respectively (p<0.01) (Fig 4A) in female monolayers compared with male. In addition, Na,K-ATPase subunit mRNA levels, α_1_-, α_2_- and β_1_-, were significantly higher in FDLE cells of female origin. The Na,K-ATPase-α_1_ subunit mRNA expression level was up to 1.4-fold higher in females (p<0.001) (Fig 4B), while the Na,K-ATPase-β_1_ subunit was 1.8-fold higher compared with males (p<0.01) (Fig 4B). The expression level of the Na,K-ATPase-α_2_ subunit was approximately 1000 times lower than that of the α_1_-subunit, but was also 1.4-fold higher in females compared with males (p<0.001) (Fig 4B). Therefore, mRNA expression analysis confirmed the higher Na^+^ currents observed in Ussing chamber measurements.

**Fig 5:**
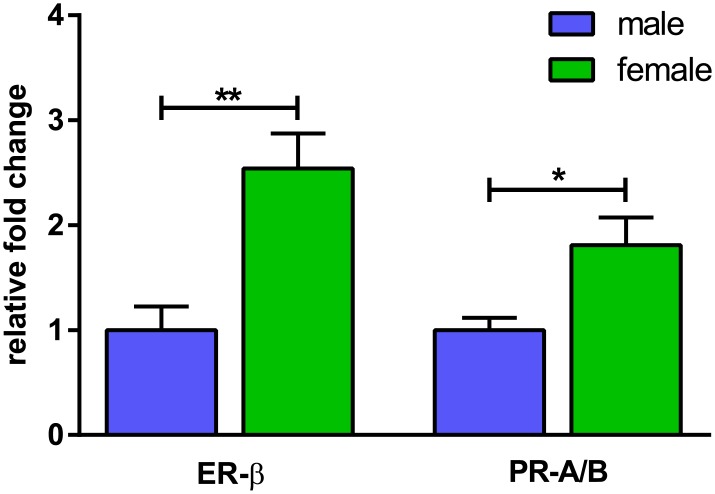
mRNA expression of ER-β and PR-A/B is higher in female FDLE cells. mRNA expression of ER-β and PR-A/B was significantly higher in female compared with male FDLE cells after 4 days in culture (n = 7–8; mean + SEM; *p<0.05 and **p<0.01 by T-test).

To elucidate potential mechanisms, mRNA expression of female sex steroid receptors, estrogen receptor β (ER-β) and progesterone receptor (PR-A/B), were measured after 4 days in culture. We have previously shown that FDLE cells do not express ER-α and PR-B, and products amplified with PR-A/B primers therefore represent mRNA expression of PR-A in FDLE cells [18,42,43]. The mRNA expression analysis showed significantly higher levels for ER-β (2.5-fold; p<0.01) and PR-A (1.8-fold; p<0.05) in cells of female origin (Fig 5).

**Fig 6:**
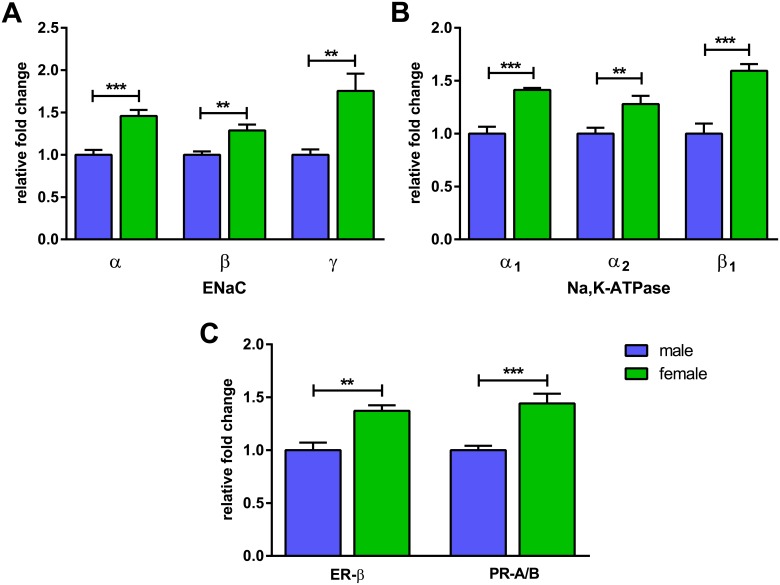
mRNA expression of ENaC and Na,K-ATPase subunits, ER-β and PR-A/B is higher in non-cultured female FDLE cells. mRNA expression determined directly after cell isolation was significantly higher in female compared with male fetuses for (A) α-ENaC, β-ENaC and γ-ENaC, (B) Na,K-ATPase-α_1_, Na,K-ATPase-α_2_ and Na,K-ATPase-β_1_, (C) ER-β and PR-A/B (n = 6–10; mean + SEM, **p<0.01 and ***p<0.001 by T-test).

To detect possible influences of cell culture conditions, mRNA expression was measured directly after cell isolation. The mRNA expression level of the ENaC subunits was also significantly higher in female FDLE cells. The α-ENaC subunit mRNA level in female cells was 1.5-fold (p<0.001), the β-ENaC subunit 1.3-fold (p<0.01) and the γ-ENaC subunit 1.8-fold (p<0.01) higher compared with male cells (Fig 6A). The Na,K-ATPase subunit levels were also higher in freshly isolated female cells, with the α_1_-subunit 1.4-fold (p<0.001), the α_2_-subunit 1.3-fold (p<0.01) and the β_1_-subunit 1.6-fold (p<0.001) higher than in male cells (Fig 6B). Finally, higher mRNA expression of female sex steroid receptors in female FDLE monolayers was confirmed by a higher level of ER-β (1.4-fold; p<0.01) and PR-A (1.4-fold; p<0.001) directly after cell isolation (Fig 6C). Therefore, mRNA expression patterns in freshly isolated cells confirmed the results obtained after 4 days in culture.

### Involvement of ER-β

**Fig 7:**
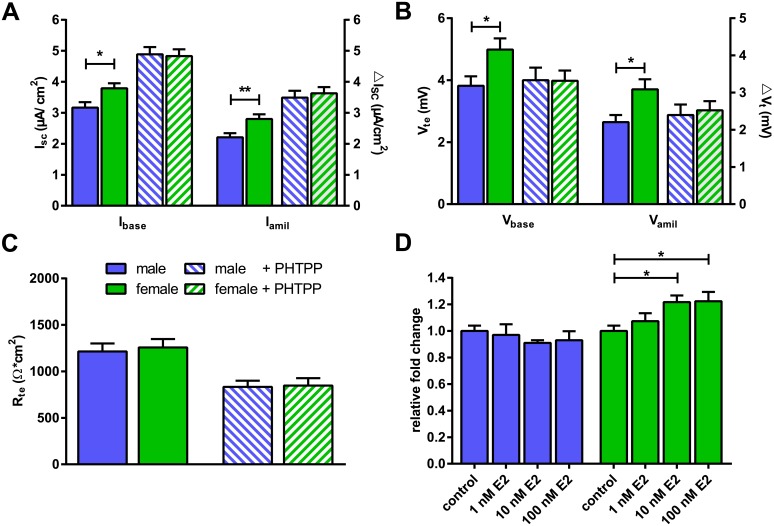
Inhibition of ER-β abolishes the sex differences in Na^+^ transport and female FDLE cells are more responsive to E2 stimulation. (A) I_base_ and I_amil_ were significantly higher in FDLE cells of female origin, while PHTPP abolished the sex difference (n = 23–28; mean + SEM; *p<0.05; **p<0.01 by T-test). (B) V_base_ and V_amil_ were significantly higher in FDLE cells of female origin, while PHTPP abolished the sex difference (n = 23–28; mean + SEM; *p<0.05 by T-test). (C) PHTPP decreased R_te_, but no R_te_ differences were observed between male and female FDLE cells. (D) PDGF-A mRNA expression in response to different E2 concentrations in male and female FDLE monolayers. Female cells are more responsive to E2 since PDGF-A mRNA expression was significantly increased in females, whereas PDGF-A mRNA expression was not affected by E2 in male FDLE cells. (♂: relative difference to male control; ♀: relative difference to female control; n = 6; mean + SEM; *p<0.05 by ANOVA with Dunnett’s *post hoc* test).

To determine an involvement of ER-β in the sex differences observed in Na^+^ transport, FDLE cells were incubated with 20 μM PHTPP. Incubation with PHTPP abolished the sex differences between male and female monolayers since no significant differences were observed for I_base_ (♂: 4.9 ± 0.2 and ♀: 4.8 ± 0.2 μA/cm^2^; p>0.05) and I_amil_ (♂: 3.5 ± 0.2 and ♀: 3.6 ± 0.2 μA/cm^2^; p>0.05) (Fig 7A). In contrast, control monolayers incubated without PHTPP demonstrated a significantly higher I_base_ (♂: 3.2 ± 0.2 to ♀: 3.8 ± 0.2 μA/cm^2^; p<0.05) and I_amil_ (♂: 2.2 ± 0.1 to ♀: 2.8 ± 0.2 μA/cm^2^; p<0.01) in female derived monolayers (Fig 7A). Likewise, V_base_ (♂: 3.8 ± 0.3 to ♀: 5.0 ± 0.4 mV; p<0.05) and V_amil_ (♂: 2.7 ± 0.2 to ♀: 3.7 ± 0.3 mV; p<0.05) were significantly higher in female FDLE cells incubated without PHTPP compared with male (Fig 7B). Furthermore, no significant differences in V_base_ (♂: 4.0 ± 0.4 and ♀: 4.0 ± 0.3 mV; p>0.05) or V_amil_ (♂: 2.9 ± 0.3 and ♀: 3.0 ± 0.3 mV; p>0.05) were observed between male and female monolayer in the presence of PHTPP (Fig 7B). Since PHTPP decreased R_te_ (Fig 7C), I_SC_ of PHTPP-incubated monolayers resulted in higher values, whereas V_te_ of PHTPP-incubated monolayers was equal to control monolayers without PHTPP.

Responsiveness towards estrogen was determined by measuring platelet-derived growth factor A (PDGF-A) mRNA expression. An increase of PDGF-A mRNA expression was observed with E2 concentrations as low as 10 nM in female derived FDLE cells (1.2-fold; p<0.05), whereas PDGF-A expression levels did not increase with E2 concentrations up to 100 nM in males (Fig 7D).

### FDLE cell number

**Fig 8:**
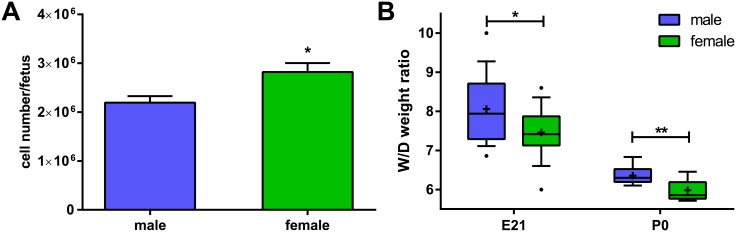
FDLE cell number per fetus is higher and lung W/D weight ratio lower in female rat pups. (A) FDLE cell numbers were significantly higher in females (n = 13 cell isolations; mean + SEM; *p<0,05 by T-test). (B) Lung W/D weight ratio was significantly lower in female fetal rats (n = 18/18; mean + SEM, *p<0.05 by T-test) and female newborn rats (n = 9/9; mean + SEM, **p<0.01 by T-test).

For comparison of FDLE cell numbers in female fetal rat lungs compared with male lungs, cell numbers obtained from 13 different cell isolations were used. The resulting FDLE cell numbers per fetus were significantly higher in female fetal lungs than in males (♂: 2.2*10^6^ ± 0.1*10^6^ to ♀: 2.8*10^6^ ± 0.2*10^6^ FDLE cells/fetus; p<0.05) (Fig 8A).

### Lung W/D weight ratio

Lung W/D weight ratio, an index of lung water content, was determined in fetal and newborn rat pups. Female pups demonstrated significantly lower lung W/D weight ratios compared with males. In fetal rats (E21) lung W/D weight ratio in males was 8.1 ± 0.2 compared to females with 7.5 ± 0.1 (p<0.05) (Fig 8B). In newborn rats (P0) lung W/D weight ratio was lower compared with fetal rats. Furthermore, males had a higher lung W/D weight ratio of 6.4 ± 0.1 compared to females with 6.0 ± 0.1 (p<0.01) (Fig 8B).

## Discussion

It has been known for almost 30 years that male preterm infants have a higher risk for developing pulmonary complications compared with female infants of the same gestational age [2]. The clinical use of antenatal steroids and surfactant replacement therapy reduced the risk for pulmonary complications in preterm infants yet the gender imbalance for respiratory distress still exists as current studies repeatedly showed [14,15]. This emphasizes the need for sex-specific studies to overcome the impairments associated with male gender. Elucidating the mechanisms of the gender imbalance may lead to better pathophysiological insights and possibly new therapies to reduce morbidity and mortality of RDS for both genders alike.

Causes of the male disadvantage in RDS incidence and severity still remain elusive. Some studies demonstrated aspects that might, in part, account for a gender imbalance. To our knowledge, this is the first study demonstrating that preterm females have a functional advantage with regard to alveolar epithelial Na^+^ transport. Our study shows that female fetal alveolar cells of the saccular stage of lung development have a higher Na^+^ transport activity compared to age matched male cells. Ussing chamber measurements demonstrated a significantly higher baseline I_SC_ as well as a higher amiloride-sensitive I_SC_ in FDLE cells of female origin. Since Na^+^ transport driven AFC is crucial for postnatal adaptation and survival at birth, the results suggest that perinatal lung transition to air breathing is superior in females.

In accordance with our results, a functional sex-related dimorphism in AFC has been shown in adult patients with acute lung injury [44], although the mechanisms by which AFC was elevated in female patients have not been elucidated. In addition, an increased AFC has been demonstrated in adult female rats, which was abolished by bilateral ovariectomy [45].

We further demonstrated a higher Na^+^ transport activity across both the apical and the basolateral membrane in measurements in which the opposite membrane was permeabilized. These experiments showed that the cells’ sex affects ENaC in the apical and also the Na,K-ATPase at the basolateral membrane. Therefore, female sex is associated with a higher activity of the whole Na^+^ transport machinery, including ENaC and the Na,K-ATPase.

To elucidate the mechanism by which the higher Na^+^ transport in females is achieved, we measured the mRNA expression of the involved Na^+^ transporters. Results demonstrated a markedly higher mRNA level of all three ENaC subunits and also of Na,K-ATPase-α_1_, -α_2_ and -β_1_ subunits in FDLE cells of female origin. Prior studies demonstrated that the α-ENaC subunit is indispensable for a functional channel and, in contrast to β- and γ-ENaC, α-ENaC is able to build a functional channel by itself. Necessity of α-ENaC was confirmed in α-ENaC knock out mice which died shortly after birth due to defective AFC [9]. Furthermore, these α-ENaC knock out mice also demonstrated the crucial role ENaC plays in perinatal lung adaptation to air breathing. However, channels consisting of α-ENaC alone only achieve about 1% of the maximal whole-cell current [46]. The β- and γ-ENaC subunits, expressed alone or in combination, are not able to induce any current, yet they markedly increase the current when co-expressed with α-ENaC [46,47]. Thus, the higher expression of all three ENaC subunits is required to support the higher currents measured in intact cells as well as basolaterally permeabilized cells. Likewise, the higher expression of the Na,K-ATPase α_1_-, α_2_- and β_1_-subunits explains the higher ouab_max_ in female cells, because these subunits are sufficient to build functional units [8], whereby the smaller β_1_-subunit is rate-limiting for Na,K-ATPase membrane insertion and activity in epithelial cells [48,49].

Since mRNA expression patterns, showing higher levels of ENaC and Na,K-ATPase in females, were similar directly after cell isolation and after 4 days in culture, culture conditions are probably not responsible for the observed sex differences. Moreover, differences in cell number do not account for the observed sex differences as equal numbers for both genders were seeded on permeable membranes for Ussing chamber and mRNA analyses. R_te_ of male and female monolayers did also not differ and for direct RNA isolation equal cell numbers were lysed.

We did not perform Western Blot analyses of Na^+^ transporter protein expression which is a limitation of this study. Therefore, we cannot exclude that posttranslational effects, in addition to elevated mRNA expression, might also contribute to the higher Na^+^ transport in female FDLE cells. Furthermore, differences in Na^+^ transport could also arise from an increased channel open probability, open time or a reduced close time, which has to be determined in future studies.

The reason for the higher expression and activity of Na^+^ transporters in females is yet unknown. Steroids have been shown to influence Na^+^ transport in alveolar epithelial cells. Both female sex hormones [18,25] and glucocorticoids [19,20] increase Na^+^ transport and mRNA expression of the facilitating Na^+^ transporters. E2 increases ENaC channel gating and plasma membrane density through G-protein coupled ER in a rat alveolar cell line [17], and elevates apical plasma membrane abundance of γ-ENaC, mediated by stimulation of protein kinase C delta (PKCδ), via ER in a kidney cell line [50]. Another study showed that E2 binding to ER-β increased expression of N-myc downstream-regulated gene 2 (NDRG2). NDRG2 interacts with Na,K-ATPase-β_1_ and stabilizes the β_1_-subunit by inhibiting its degradation thereby increasing Na,K-ATPase-mediated Na^+^ transport [51]. Furthermore, prenatal inhibition of ER and PR reduced the amiloride-sensitive AFC in newborn piglets [21]. However, the levels of female sex steroids and glucocorticoids during fetal development are equal for both genders as they are determined by the placenta [52]. We did not determine steroid concentrations in the FBS-supplemented cell culture media presumably containing cortisol, female sex steroids and androgens, but serum levels were not different between male and female cells during cell culture and RNA isolation directly after cell isolation confirmed our results. Therefore, steroid concentrations *per se* are unlikely the cause for the observed gender differences. However, a different responsiveness to equal steroid concentrations in the cell culture medium and also in the fetal circulation might contribute to sex differences. Since FDLE cells do not express ER-α we focused on ER-β whose expression in FDLE cells has been demonstrated before [18,42]. Furthermore, we analyzed the expression of PR in FDLE cells which have been shown to express PR-A only [43]. Results demonstrated higher mRNA levels for ER-β and PR in female FDLE cells, both directly after cell isolation and after 4 days in culture. In concert with our results, a similar sexual dimorphism for PR and ER-β expression was demonstrated in adult rats [53]. Inhibition of ER-β with PHTPP abolished the sex differences in Na^+^ transport, further supporting a critical involvement of ER-β. Moreover, PDGF-A mRNA expression was more responsive to E2 in female-derived FDLE cells. PDGF-A represents a key regulator of alveolar formation and its gene expression is regulated by estrogen via ER-β in the lung [42,54], making PDGF-A a suitable ER-β target gene to determine estrogen responsiveness in alveolar cells. In conclusion, our results demonstrate that ER-β is crucially involved in the sex differences observed in alveolar Na^+^ transport. Furthermore, higher abundance of female sex steroid receptors possibly renders female FDLE cells more responsive to steroids *in utero* and to serum components, and might therefore account for the higher Na^+^ transport observed in our measurements.

Female sex steroid receptors might not be the only factor contributing to the sex difference in Na^+^ transport since androgens have been shown to delay fetal lung maturation [55,56]. In particular, the male delay in surfactant production, known as a causative factor for RDS development [2,3], underlies regulation by androgens [13,57–59]. Few studies addressed the influence of androgens on Na^+^ transport. In ovariectomized female rats, androgen injection has been shown to decrease the expression of all ENaC subunits in the kidney [60]. In contrast, testosterone increased the mRNA expression of α-ENaC in the human renal cell line HKC-8 [61]. Furthermore, a putative androgen response element was identified in the α-ENaC promoter region, suggesting transcriptional regulation by androgens [60]. Thus, a regulation of ENaC by androgens, naturally elevated in male fetuses, might be assumed; however, the direction of this putative regulation remains inconclusive and has not been addressed in the lung.

Several microarray studies have analyzed sex-dependent gene expression in fetal rodent lungs [62,63]. It was shown that co-regulators of the androgen receptor (AR) are up-regulated in the male murine fetal lung of the saccular stage [62]. The study also demonstrated that the male lung of the early saccular stage is still engaged in proliferative pathways while the female lung already pursues lung maturation [62]. In addition, sex-dependent expression differences in genes related to metabolism and transport of steroid and non-steroid hormones like thyroid hormones were described [63]. Sex differences in AR interacting genes and steroid hormone metabolism supports the view that sex steroid pathways are responsible for the male delay in lung maturation yet putative differences in metabolism and transport of other hormones might also be involved. A variety of hormones regulate Na^+^ transport and lung maturation including thyroid hormones [64] which also showed sex-specific expression patterns [63] and might therefore contribute to differences in Na^+^ transport.

In addition to the higher alveolar epithelial Na^+^ transport demonstrated in premature females, another sexual dimorphism was observed in our study. The FDLE cell number per fetus was approximately 30% higher in females compared to males. Sex differences in the architecture of lung's gas exchange regions of adult rats and mice have been described [65]. In particular, female rodents were shown to possess smaller and more alveoli per body mass compared to male rodents, resulting in a larger gas-exchange area per body mass in females [65,66]. We were able to confirm the sexual dimorphism of alveolar cell number, yet in premature rodents. Studies in ovariectomized rats showed that these animals had larger alveoli and a lower alveolar number with a smaller gas-exchange area, which was prevented by E2 treatment [67,68]. Furthermore, E2-treated sham-ovariectomized rats had smaller and more numerous alveoli than untreated controls and prenatal ER/PR inhibition reduced alveolar counts in newborn piglets [21]. These observations are not able to explain the differences in FDLE cell number in our study, because estrogen concentration is determined by maternal hormone levels during pregnancy [52]. However, our study also revealed a higher level of ER-β in female fetuses which might account for the observed higher alveolar cell number. Experiments with ER-β knockout mice showed a decreased number of alveoli in females while impact on male animals was marginal [42,66]. Thus, expression of ER-β affects alveolar number and might therefore be responsible for the higher FDLE cell number in females.

Studies showed that AFC is directly related to alveolar Na^+^ transport [9,69] and higher Na^+^ transport supposedly increases AFC. Clearance of lung liquid begins well before the onset of labor [70–73] and Na^+^ transport across the pulmonary epithelium begins in late gestation, since amiloride treatment of the near-term fetus increased the rate of secretion [74]. These studies suggest that Na^+^ transport increases as term approaches so that the absorptive and secretory processes are almost in balance with the onset of labor [70]. We therefore measured lung water content of fetal rats retrieved approximately 24–48 h prior to birth and newborn rat pups. Lung water content was significantly lower in female fetal and newborn rats compared with male. The lower lung water content together with the higher Na^+^ transport suggests a higher rate of AFC in females or a more advanced onset of absorptive processes.

Concluding, we showed a higher vectorial Na^+^ transport in FDLE cells of premature females, possibly attributable to a higher mRNA expression of ENaC and Na,K-ATPase that might be mediated by an increased responsiveness to female sex steroids via higher expression of ER-β and PR. The sex-dependent differences in Na^+^ transport were corroborated by a larger amount of FDLE cells per fetus and a lower lung water content in females compared with males. The study therefore showed a functional advantage for the female sex, and a possible mechanism, which may contribute to the gender imbalance observed in occurrence and outcome of RDS in preterm infants.
